# *Bifidobacterium lactis*-Derived Vesicles Attenuate Hippocampal Neuroinflammation by Targeting IL-33 to Regulate FoxO6/P53 Signaling

**DOI:** 10.3390/nu16213586

**Published:** 2024-10-22

**Authors:** Xiaoyu Du, Ming Zhang, Ran Wang, Zhaozhong Zeng, Wen Zhao, Bing Fang, Hanglian Lan, Weilian Hung, Haina Gao

**Affiliations:** 1 School of Food and Health, Beijing Technology and Business University, Beijing 100048, China; 18223846930@163.com (X.D.); zhangming@th.btbu.edu.cn (M.Z.); 2 Key Laboratory of Precision Nutrition and Food Quality, Department of Nutrition and Health, China Agricultural University, Beijing 100083, China; wangran@cau.edu.cn (R.W.); zhaowen@nctid.cn (W.Z.); bingfang@cau.edu.cn (B.F.); 3 National Center of Technology Innovation for Dairy, Hohhot 010110, China; cengzhaozhong@yili.com (Z.Z.); lanhanglian@yili.com (H.L.); 4 Inner Mongolia Yili Industrial Group Co., Ltd., Hohhot 010100, China

**Keywords:** BL99, microbiota-derived vesicles, hippocampal neuroinflammation

## Abstract

Background: Hippocampal Neuroinflammation (HNF) is a critical driver of cognitive impairment. The lipopolysaccharide (LPS) accumulate amyloid beta (Aβ) and lead to HNF. The *Bifidobacterium lactis* (BL) 99 have anti-inflammatory ability. However, whether BL99-derived microbiota-derived vesicles (MV) could alleviate LPS-induced HNF remains unclear. Methods: To investigate, we used ultrafiltration with ultracentrifuge to extract BL99-derived-MV (BL99-MV). We used hippocampal neuronal HT22 cells (HT22) to establish the LPS-induced HNF model, and explored whether BL99-MV alleviate LPS-induced HNF. Results: The confocal microscopy showed that BL99-MV were taken up by HT22 and reduced the oxidative stress (ROS) level. The PCR showed that BL99-MV up-regulate IL-10 level, and down-regulate TNF-α, IL-1β, and IL-6. Transcriptomic analysis revealed 4127 differentially expressed genes, with 2549 genes upregulated and 1578 genes downregulated in the BL99-MV group compared to the LPS group. Compared to the LPS group, BL99-MV decreased FoxO6, IL-33, P53, and NFκB expression, but increased FoxO1 and Bcl2 expression. The WB showed that BL99-MV modulated NFκB, FoxO6, P53, Caspase9, and Caspase3 protein expression by reducing IL-33 expression in HT22. The findings demonstrated IL-33 as a regulator for FoxO6/P53 signaling. Conclusions: Here, we hypothesized that BL99-MV alleviated LPS-induced HNF to promote HT22 survival and synaptic development by regulating FoxO6/P53 signaling by targeting IL-33.

## 1. Introduction

The prevalence of neuroinflammation has increased following the onset of COVID-19, with affected patients experiencing high morbidity and mortality [1,2]. Neuroinflammatory disorders, including depression, anxiety-like disorders, and cognitive and memory impairments, have become more common [3,4]. The hippocampus, essential for learning and memory, is one of the initial brain regions to be impacted, making hippocampal neuroinflammation (HNF) a significant contributor to these disorders [5,6]. The hippocampal neuronal HT22 cells (HT22) act as an important model whose function can be impaired upon induction by LPS-induced HNF [7]. When lipopolysaccharide (LPS) enters the central nervous system (CNS), it activates Toll-like receptor 4 (TLR4), which can trigger HNF and promote the secretion of amyloid-beta (Aβ) peptides [8,9,10,11]. This process is driven by neuroimmune cells (microglia and astrocytes), which enter a proinflammatory state and release cytokines such as Interleukin-33 (IL-33), Interleukin-1β (IL-1β), Interleukin-6 (IL-6), Interleukin-10 (IL-10), Tumor Necrosis Factor-α (TNF-α), and reactive oxygen species (ROS). These factors contribute to synaptic dysfunction, synapse loss, and reduced survival of HT22 during HNF [12,13].

The IL-33 is produced in various tissues and highly expressed in the CNS, especially within the hippocampus where it is predominantly produced by neurons, and it emerges as a key player in HNF [14]. Research indicates that IL-33 has been shown to have the potential to impede the onset of Alzheimer’s disease in models conducted both in vitro and in vivo [15]. The elevated expression of IL-33 in cell models has been shown to specifically reduce the production of Aβ peptides [16]. After proinflammatory triggers, IL-33 is secreted into the extracellular space and attaches to its specific receptor, IL1RL1 [17]. This interaction triggers the mitogen-activated protein kinases and NFκB signaling pathways. These pathways contribute to the formation of Aβ, thereby inducing HNF and oxidative stress [18,19].

Forkhead box protein O1 (FoxO1) and Forkhead box protein O6 (FoxO6) belong to the Forkhead box protein O class, which are critical to regulate oxidative stress and inflammation within brain [19]. Although FoxO1 is broadly distributed and expressed across various tissues, FoxO6 expression is predominantly limited to neural cells [20]. It has been found that FoxO1 mediates immune responses by influencing the release of inflammatory factors [21]. Hippocampal neuron dendritic spine density is associated with FoxO6, which is essential for memory encoding [22]. Notably, FoxO1 predominantly localizes in the cytosol, whereas FoxO6 exhibits high nuclear localization [23]. This nuclear presence of FoxO6 is crucial for its ability to modulate the intracellular redox state. By remaining in the nucleus, FoxO6 can react sensitively to both external and intracellular stimuli, thereby regulating antioxidant gene expression [24]. Therefore, FoxO6 is crucial to various cellular processes, including cellular survival, proliferation, cell death, metabolic activity, and aging [25]. In addition, the tumor protein p53 (p53), a marker of apoptosis, which is activated in response to LPS-induced oxidative stress, can act as a transcription factor regulating cell viability and promoting the expression of various genes [26].

The crucial role probiotics play in modulating HNF and neurodegeneration via the gut–brain axis is increasingly recognized [27,28,29,30,31]. As an illustration, *Akkermansia muciniphila* has been shown to promote hippocampal neurogenesis and improve neuroinflammation by activating the brain-derived neurotrophic factor signaling pathway [32]. Similarly, *Lactobacilli* and *Bifidobacteria* have proven beneficial in improving cognitive deficits in the Aβ model [33]. Additionally, *Lactobacillus helveticus* reduced ampicillin-induced spatial memory deficits and enhanced spatial memory performance under continuous restraint stress by increasing levels of the cytokine involved in anti-inflammatory responses and restoring brain-derived neurotrophic factor levels [34]. Administering *Lactobacillus* to mice has also been shown to ameliorate synaptic loss and mitochondrial dysfunction in the brain [35]. Recent research has shown that *Bifidobacterium* improves memory and prevents brain atrophy in populations with cognitive impairment. *Bifidobacterium breve HNXY26M4* supplementation generates acetate and butyrate, strengthens the blood–brain barrier (BBB), and alleviates Aβ deposition, cognitive impairment, neuroinflammation, and synaptic impairment [36]. *Bifidobacterium breve MCC1274* cell extracts prevent brain inflammation by blocking the perilipin 4 expression to reduce lipid droplet formation [37]. The *Bifidobacterium lactis* (BL)99 is a Gram-positive bacterium, and its abundance is inversely correlated with several diseases, such as functional dyspepsia, osteoporosis, and colitis [38,39,40]. However, whether the BL99-derived microbiota-derived vesicles (MV) could alleviate LPS-induced HNF remains unclear.

Recent studies have been identified that MV bilayered membrane nanostructures ranging from 50 to 400 nm in size [41]. These vesicles carry bioactive cargo and facilitate communication between microbes and the host [42]. Interestingly, MV could be taken up by neurons, which, in turn, improves neuroinflammation and alleviates cognitive impairment [43,44,45]. *Lactobacillus paracasei*-derived MV directly influence transcriptional responses to Aβ and Aβ-linked pathology conditions in the brain [46]. *Lactobacillus* species-derived MV have been shown to alleviate neurocognitive dysfunction by decreasing the release of IL-1β and IL-6, and increasing IL-10 production following LPS stimulation [47]. Similarly, *Lactobacillus plantarum*-derived MV alleviate oxidative stress biomarkers and cytokines involved in inflammation in both the brain and serum [48]. Furthermore, MV from *Propionibacterium freudenreichii* can modulate the NFκB pathway to exhibit inflammatory-modulating effects and engage in interactions with the host that modulate immune responses through the surface protein SlpB [49]. This suggests that MV can selectively target CNS cells, potentially playing a critical role in microbe–microbe and host–microbe interactions. Our study aimed to identify the effect of BL99-derived-MV (BL99-MV) on LPS-induced HT22 inflammatory injury. To this end, we might clarify the protective effect of BL99-MV on HT22 under LPS-induced HNF to provide a theoretical foundation for early intervention strategies.

## 2. Materials and Methods

### 2.1. Bacterial Strains

The BL99 was prepared as a powder at 1.5 × 10^11^ CFU g^−1^ by Yili Co., Ltd. (Hohhot, China) [39]. BL99 was anaerobically cultured in an MRS medium (cat. #CM187, Land bridge) with a supplement of 0.05% L-cysteine (Biotopped, Beijing, China) before being harvested at 37 °C overnight.

### 2.2. BL99-MV Isolation

The bacterial suspensions (2.24 × 10^8^ CFU/mL) were centrifuged for 30 min (3600× *g*, 4 °C) [50]. Cells were pelleted via centrifugation at 5000× *g*, and emptied into a suitable centrifuge bottle [51]. Subsequently, the supernatants were centrifugated for 60 min (11,000× *g*, 4 °C) to eliminate debris [52]. The supernatants obtained were used for subsequent experiments by Method 1 and Method 2. In Method 1 (ultracentrifuge), the supernatant was ultracentrifuged for 90 min (120,000× *g*, 4 °C) [53]. The pelleted BL99-MV were re-suspended in PBS and then ultra-centrifuged for washing using an SW 8T rotor. Finally, the BL99-MV were passed through 0.22-μm filters, and then re-suspended in PBS and stored in a freezer at −80 °C until use [54]. In Method 2 (ultrafiltration and ultracentrifuge), the supernatant was concentrated 10-fold using 100-kDa ultrafiltration tubes (Millipore, Burlington, NJ, USA) for 10 min (5000× *g*, 4 °C) and ultracentrifuged for 90 min (120,000× *g*, 4 °C) [55]. The pelleted BL99-MV were resuspended in PBS and then ultra-centrifuged for washing using an SW 8T rotor. Finally, the BL99-MV were passed through 0.22-μm filters, and then re-suspended in PBS and stored in a freezer at −80 °C until use.

### 2.3. BL99-MV Characteristics

We divided the BL99 growth process into four stages: lag phase, logarithmic phase, early stationary phase, and late stationary phase to maintain it at 37 °C for 36 h, and optical density at 600 nm (OD600) was measured [38].

#### 2.3.1. Transmission Electron Microscopy (TEM)

The BL99-MV in deionized water were set on a 300-mesh nickel grid and allowed to dry for 30 min. The excess solution was removed with filter paper. The grid was stained with 2% uranyl acetate in water for 10 min and washed by transferring it onto several drops of deionized water, incubating it overnight, and drying it further. The grid was then analyzed using a JEOL JEM1200EX TEM (JEOL, Tokyo, Japan) [54].

#### 2.3.2. Size Analysis

Particle size distribution was measured using a Zeta-sizer Nano-series instrument (Malvern Nano-ZS, Malvern, UK) at 4 °C. The BL99-MV were diluted 500 times with PBS. The size data refer to the scattering intensity distribution [54].

#### 2.3.3. Bicinchoninic Acid Assay (BCA)

Protein content was measured using the BCA Protein Assay Kit (Solarbio, Beijing, China). All procedures were performed according to the manufacturer’s instructions, and protein content was determined by comparing it with a BCA standard curve [56].

#### 2.3.4. Flow Cytometry (FC)

The BL99-MV were characterized and quantified using a Red Fluorescent Cell Linker Kit for General Cell Membrane Labeling (PKH26) (Sigma-Aldrich, Shanghai, China) and analyzed via an intensity plot. The relative labeled amount of PKH26 to MV determined the characterization and concentration of the BL99-MV per milliliter. Purified exosomes were labeled with a yellow-orange fluorescent dye with PKH26. The BL99-MV were mixed in Diluent C (at a concentration of 200 ng/μL). Separately, PKH26 was combined with Diluent C (1:250). The BL99-MV resuspension solution was combined with the PKH26 working solution and incubated. The labeling reaction was terminated by adding 5 mL of 1% BSA, followed by washing with PBS. The same concentration of PKH26 was centrifuged in parallel to create a background control. Acquisition was performed using the Image Stream-X Imaging FC (Amnis Corporation, Seattle, WA, USA) equipped with INSPIRE software. A minimum of 300,000 BL99-MV were analyzed for each sample. IDEAS software was used for data analysis (Amnis Corporation) [54].

### 2.4. Cell Cultures and Treatment

The HT22 were obtained from iCell (Bioscience Inc., Shanghai, China). The cells were cultured in 60 mm dishes (SORFA, Zhejiang, China) incubated under normoxic conditions. Dulbecco’s modified Eagle medium (Gibco, Brisbane, Australia) containing 10% fetal cow serum (Tianhang, Zhejiang, China) was used as the basal growth medium until 80% confluence was reached. The cells were then passaged with 0.25% trypsin-0.02% EDTA (Beyotime, Jiangsu, China) [57]. Afterward, the cells were serum-starved overnight, the cells were divided into three groups: the Ctrl group, the LPS group, and the BL99-MV group. The Ctrl group growth was collected. The LPS group was replaced with LPS (1 mg/mL) (Sigma-Aldrich, Shanghai, China) for 24 h to induce HNF. The BL99-MV group was replaced with BL99-MV protein concentrations of 50, 100, 200, and 400 ng/μL working solution for 12 h, then transferred into LPS for 24 h.

#### 2.4.1. Cell Counting Kit-8 Assay

Logarithmic phase cells were harvested and seeded in 96-well culture plates. When the cells grew to about 80%, they were classified into a control (Ctrl) group, an LPS group (1 mg/mL), and a BL99-MV group (*n* = 3). The BL99-MV (50, 100, 200, and 400 ng/μL) were pretreated for 6 h, 12 h, and 24 h in HT22 before being treated with LPS for 24 h. Experiments were carried out strictly according to the manufacturer’s instructions (Seven Sea Biotech, Shanghai, China). Briefly, CCK-8 solution was added to the medium in the wells of a 96-well cell culture plate and incubated at 37 °C for 2 h. Absorbance at 450 nm was measured using a microplate reader (BioTek, Vermont, USA) [58].

#### 2.4.2. Immunofluorescence (IF)

##### Uptake of BL99-MV by HT22

The BL99-MV were labeled with a PKH26, according to the manufacturer’s instructions. The BL99-MV were resuspended in Diluent C (1:5). Separately, PKH26 was mixed with Diluent C (1:250). The BL99-MV resuspension solution was combined with the PKH26 working solution and incubated. After labeling, 5 mL of 1% BSA was added to stop the reaction, and the sample was washed with PBS. Using the same ratio as before, PKH26 was centrifuged in parallel as a background control. The HT22 were incubated with PKH26-labeled BL99-MV working solution (at a concentration of 200 ng/μL) or the same volume of the PKH26-PBS control, and incubated serum-free for 12 h at 37 °C. After incubation, the cells were incubated with LPS for 24 h, and using 100 nM 4′,6-diamidino-2-phenylindole (DAPI) (Sigma-Aldrich, Shanghai, China) solution for 5 min. The cells were washed three times following the removal of the medium and fixative, as well as after the penetration and incubation steps. The extent of BL99-MV incorporation into the HT22 was analyzed using confocal microscopy (Zeiss, Oberkochen, Germany) [54].

##### Effect of BL99-MV on Morphological Changes of HT22

The cytoskeleton was stained using a phalloidin kit (Thermo, Waltham, MA, USA). After removal of the cell medium, cells were fixed with 4% paraformaldehyde (10 min). After removal of the fixative, cells were fixed with 0.5% Triton X-100 solution (10 min). Phalloidin working solution was then applied to completely cover the cells and they were incubated in the dark (30 min). Cell nuclei were stained with DAPI solution (100 nm) for 5 min. After staining, the cells were washed three times to remove the residual medium, fixative, and staining solutions. Cell morphology was examined using confocal microscopy.

##### ROS Assay

The HT22 cultures were incubated with DCFH-DA (10 μM) for 30 min to measure ROS. Following this, the cells were stained with DAPI in the dark (15 min, 37 °C). The cells were rinsed with PBS, and confocal microscopy was used [59].

#### 2.4.3. RNA-Seq Analysis

##### mRNA Library Construction and Sequencing

The total RNA of HT22 was extracted using Trizol Reagent (Invitrogen, Waltham, MA, USA). The RNA quality was assessed by measuring the A260/A280 ratio with a Nanodrop^TM^ OneC spectrophotometer (Thermo, Waltham, MA, USA). Raw sequencing data were initially filtered with Trimmomatic (version 0.36); low-quality reads were discarded, and reads with adaptor sequence contamination were trimmed. Clean data were aligned to the HT22 reference genome from the National Center for Biotechnology Information (https://www.nih.gov, accessed on 1 October 2024) using STRA software (version 2.5.3a) with default parameters. Genes differentially expressed (GDEs) between groups were identified using the edgeR package (version 3.12.1). |log2 (fold change)| ≥ 1.8 and *p* < 0.05 were used to judge the statistical significance of gene expression differences. The Kyoto Encyclopedia of Genes and Genomes (KEGG) enrichment analysis for differentially expressed genes was implemented by KOBAS software (version: 2.1.1), with a *p*-value cutoff of 0.05 to judge statistically significant enrichment. Alternative splicing events were detected by using rMATS (version 3.2.5) with an FDR value cutoff of 0.05 and an absolute value of Δψ of 0.05 [60].

##### RNA Extraction and Quantitative Real-Time PCR

For RNA expression analysis, cDNA was synthesized using the Prime Script RT Reagent Kit with gDNA Eraser (TaKaRa, Shiga, Japan), following the manufacturer’s instructions. A quantitative real-time reverse transcriptase-polymerase chain reaction (qRT-PCR) was performed using TB Green^®^ Premix Ex Taq™ (TaKaRa, Shiga, Japan). The primer sequences used are listed below (Table 1).

Real-time PCR was conducted using the Step One^TM^ Real-time PCR System Thermal Cycling Block (ABI, Waltham, MA, USA) in a 20 μL reaction mixture containing 300 nM of each primer, 2 μL of template cDNA, and 18 μL of 2× SYBR Green I Master Mix (TaKaRa, Shiga, Japan). The qPCR protocol included an initial step at 37 °C for 15 min, followed by 2 cycles at 85 °C for 5 s each. Actin served as the endogenous control. Relative changes in gene expression between the control and treated samples were determined using the 2^−ΔΔCt^ method [43].

#### 2.4.4. Western Blot Analysis

Cell proteins were extracted using an ice-cold radioimmunoprecipitation assay (RIPA) buffer (FEIMOBIO, Beijing, China) containing 1 mM phenylmethylsulfonyl fluoride (PMSF) (Solarbio, Beijing, China). Lysates containing 25 μg of protein were loaded onto gels, separated on SDS-PAGE (CWBIO, Beijing, China) and transferred to polyvinylidene fluoride transfer membranes (PVDF) (Millipore, Burlington, USA). Membranes were incubated in non-fat dry milk powder (5%) in TBS-Tween 20 for 4 h at room temperature and overnight at 4 °C, followed by blotting with the primary antibodies (diluted 1:1000 in PBS) against proteins of Aβ, Tau, NFκB, FoxO1, FoxO6, IL-33, Bcl2, P53, Caspase3 and Caspase9. The above antibodies were all purchased from proteintech group, Inc. Stains were incubated with secondary antibody (proteintech, wuhan, Beijing) diluted 1:2000 in PBS. Immunoreactive bands were visualized using Pierce ECL Western Blot Substrate (Beyotime, Shanghai, China). The density of protein bands was analyzed using Image J2x 2.1.4.7 software (Rawak Software Inc., Stuttgart, Germany). The density of band, complemented with BL99-MV, was normalized to the β-actin control [61].

### 2.5. Statistical Considerations

Results were presented as the mean ± standard error of the mean (SEM). The data were analyzed using the one-way analysis of variance (ANOVA) followed by Tukey’s multiple comparison tests (*p* < 0.05 was considered statistically significant).

## 3. Results

### 3.1. BL99-MV Different Isolation Methods

**Figure 1:**
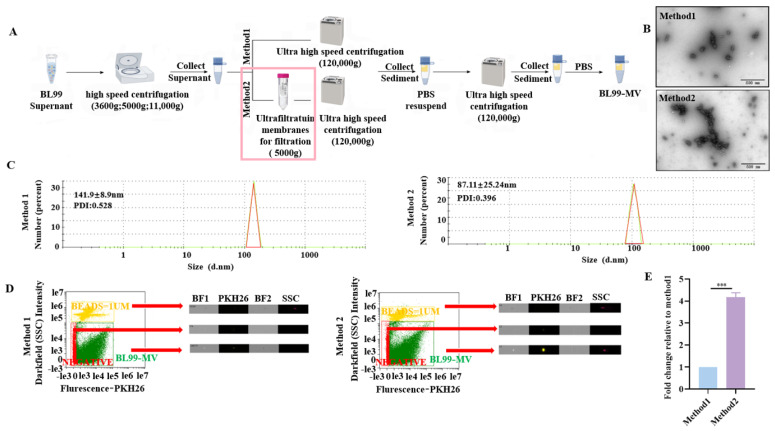
Characterization of BL99-MV by two methods. (**A**) Isolation protocol of BL99-MV. (**B**) The TEM images showing BL99-MV isolated using Method 1 and Method 2 (Scale bar: 500 nm). (**C**) The size distribution of BL99-MV isolated using Method 1 and Method 2. (**D**) Images showing the quantity and size of BL99-MV isolated using Method 1 and Method 2 were accessed by labeling with DIR. SSC = side scatter; BF1 = bright-field 1; BF2 = bright-field 2. (**E**) The relative number of BL99-MV isolated using Method 1 and Method 2. Data are presented as the mean ± SEM, with three replicates per group (*n* = 3). Means marked with asterisks are significantly different (*** *p* < 0.001).

The MV were isolated from the BL99 supernatant using two different methods (Figure 1A). Both methods successfully isolated MV, as confirmed by the results. The TEM revealed that the BL99-MV were spherical vesicles, possessing a double membrane structure (Figure 1B). The protein concentration of BL99-MV isolated by Method 2 was higher than that isolated by Method 1, as determined by the BCA assay (Supplementary Figure S1). A nanoparticle size distribution analysis showed a peak diameter of 141.9 ± 8.9 nm with a polydispersity index (PDI) of 0.528 for Method 1, and a peak diameter of 87.11 ± 25.24 nm with a PDI of 0.396 for Method 2 (Figure 1C). FC analysis indicated that Method 2 yielded a significantly higher number of BL99-MV, with a 4.17-fold increase compared to Method 1 (*p* < 0.001) (Figure 1D,E). These results suggest that Method 2 is more effective than Method 1 for the isolation of BL99-MV.

### 3.2. BL99-MV Stability Analysis

**Figure 2:**
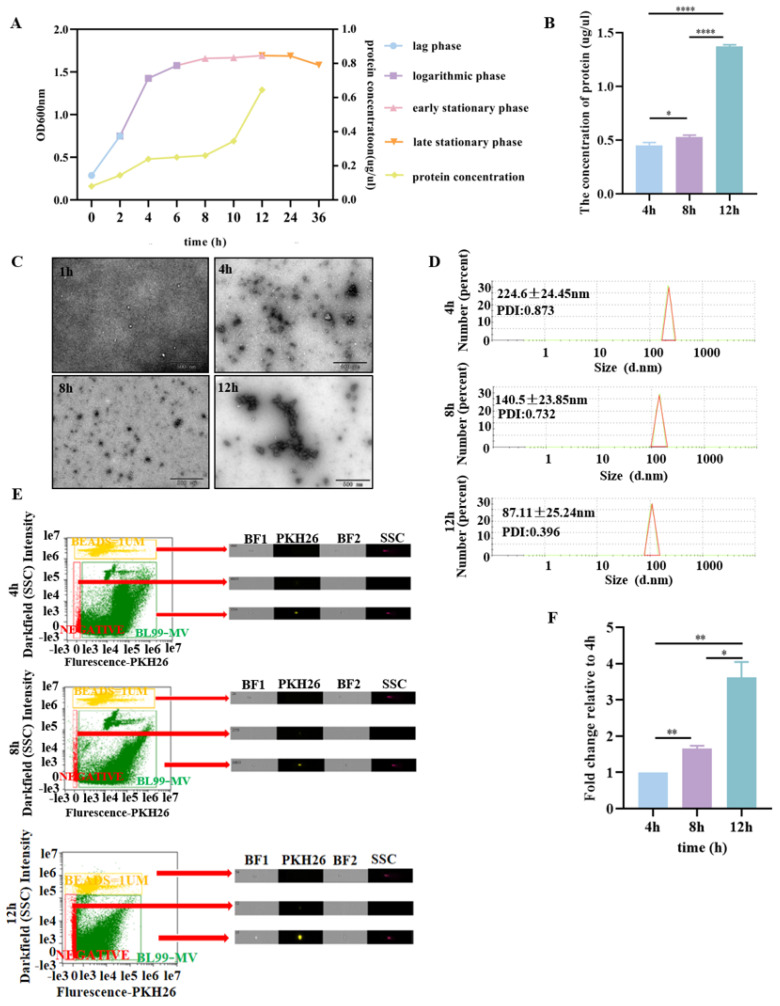
Characterization of BL99-MV at different periods. (**A**) Growth curve of BL99. (**B**) The protein concentration of MV by BCA. (**C**) Representative TEM images of BL99-MV at different periods (Scale bar: 500 nm). (**D**) Size distribution of BL99-MV analyzed via nanoparticle tracking analysis at different periods. (**E**) BL99-MV fluorescently labeled by DIR labeling, and detected in FC at different periods. SSC = side scatter; BF1 = bright-field 1; BF2 = bright-field 2. (**E**,**F**) The number of BL99-MV detected in FC at different periods. Data are presented as the mean ± SEM, with three replicates per group (*n* = 3). Means marked with asterisks are significantly different (* *p* < 0.05; ** *p* < 0.01; **** *p* < 0.0001).

To investigate the stability of BL99-MV, we isolated and analyzed MV from BL99 supernatant at different growth stages. The growth curve indicated that the 0–2 h period corresponds to the lag phase, 2–8 h to the logarithmic phase, and 8–12 h to the stationary phase. We observed that the BL99-MV protein concentration reached 1.34 µg/µL during the late stationary phase (12 h), which is a 2.68-fold increase compared to the lag phase (Figure 2A). The TEM revealed that BL99-MV were double-layer membrane-enclosed nanoparticles during both the logarithmic and stationary phases (Figure 2C). The highest protein concentration of BL99-MV, 1.34 µg/µL, was recorded during the late stationary phase, as determined by the BCA assay (Figure 2B). Nanoparticle size distribution analysis showed that BL99-MV were vesicles ranging from 30 to 400 nm. The PDI at 12 h was 0.528, indicating better dispersion compared to the samples at 2 h, 4 h, and 8 h (Figure 2D). FC analysis revealed that the highest number of BL99-MV was isolated at 12 h, with a three-fold increase compared to 4 h, and a 2.37-fold increase compared to 8 h (*p* < 0.0001) (Figure 2E,F).

### 3.3. Effect of BL99-MV Preprotection on HNF in HT22

To investigate the preprotection of BL99-MV on HNF, we determine BL99-MV effects on HT22 activity by IF, CCK-8, and qPCR, and HT22 morphological structure by IF.

#### 3.3.1. Effect of BL99-MV Preprotection on Cell Activity

**Figure 3:**
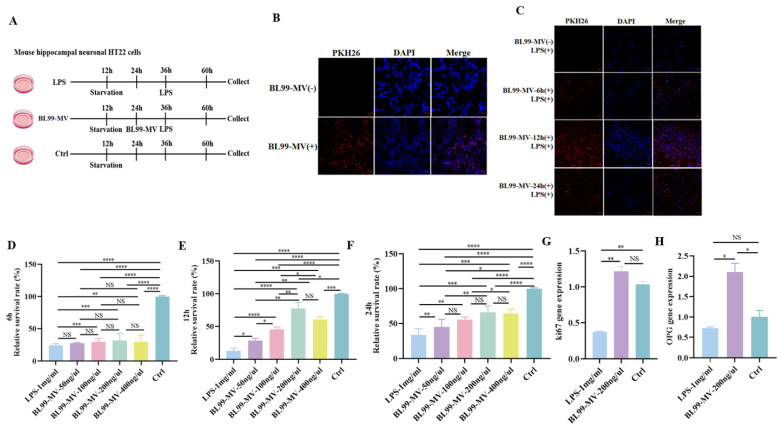
Effect of BL99-MV on HT22. (**A**) Culturing protocol of HT22. The HT22 (*n* = 3) were classified into a Ctrl group, LPS group, and BL99-MV group. (**B**) Uptake of BL99-MV by HT22. (**C**) BL99-MV on the relative survival rate (%) of HT22 by confocal microscopy. (**D**–**F**) BL99-MV on the relative survival rate (%) of HT22 by CCK-8. (**G**,**H**) The gene expression of Ki67 and OPG. Data are presented as the mean ± SEM, with three replicates per group (*n* = 3). Means marked with asterisks are significantly different (* *p* < 0.05; ** *p* < 0.01; *** *p* < 0.001; **** *p* < 0.0001; NS *p* > 0.05).

The confocal microscopy result confirmed BL99-MV’s successful incorporation into HT22 (Figure 3B). Compared to at 6 h and 24 h, cell survival was highest at 12 h (Figure 3C). The CCK-8 analysis revealed that BL99-MV significantly enhanced HT22 proliferation at concentrations of 50, 100, 200, and 400 ng/μL compared to the LPS group, with 200 ng/μL showing the most pronounced effect (*p* < 0.05). At 12 h, the 200 ng/μL concentration of BL99-MV achieved the highest cell survival, reaching up to 73%, consistent with the IF results (Figure 3D–F). Based on these findings, we selected a concentration of 200 ng/μL for treating HT22 cells for 12 h in subsequent experiments.

We measured the mRNA expression of two proliferation markers, Ki67 and OPG, in HT22 by qPCR [62]. The results demonstrated that BL99-MV significantly upregulated Ki67 and OPG mRNA expression compared to the LPS group (*p* < 0.05) (Figure 3G,H).

#### 3.3.2. Effect of BL99-MV on Cell Morphological Structure

**Figure 4:**
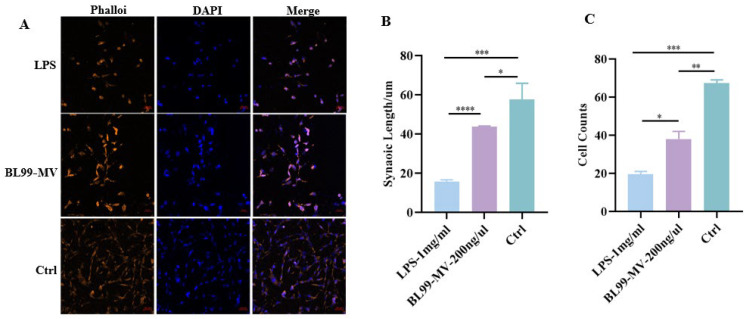
Effect of BL99-MV on HT22 Counts and Synaptic Length. (**A**) The cytoskeleton was stained with phalloidin in HT22. (**B**) The cell synaptic length in HT22. (**C**) The cell counts in HT22. Data are presented as the mean ± SEM, with three replicates per group (*n* = 3). Means marked with asterisks are significantly different (* *p* < 0.05; ** *p* < 0.01; *** *p* < 0.001; **** *p* < 0.0001).

To examine how BL99-MV affects HT22’s morphological structure, we stained the cytoskeleton with phalloidin. The results indicated that BL99-MV alleviated the damage to the cytoskeleton caused by LPS (Figure 4A). The mean synaptic length in the control group was approximately 60 µm, which decreased to about 15 µm in LPS group. In contrast, the synaptic length in the BL99-MV group was 2.86 times greater than in the LPS group (*p* < 0.0001) (Figure 4B). In the control group, the cell number was approximately 54, representing a 2.25-fold increase compared to the LPS group (*p* < 0.001). The mean number of cells in the BL99-MV group ranged between 29 and 42 (Figure 4C).

### 3.4. Effect of BL99-MV Preprotection on Anti-Oxidative and Inflammatory Cytokines

**Figure 5:**
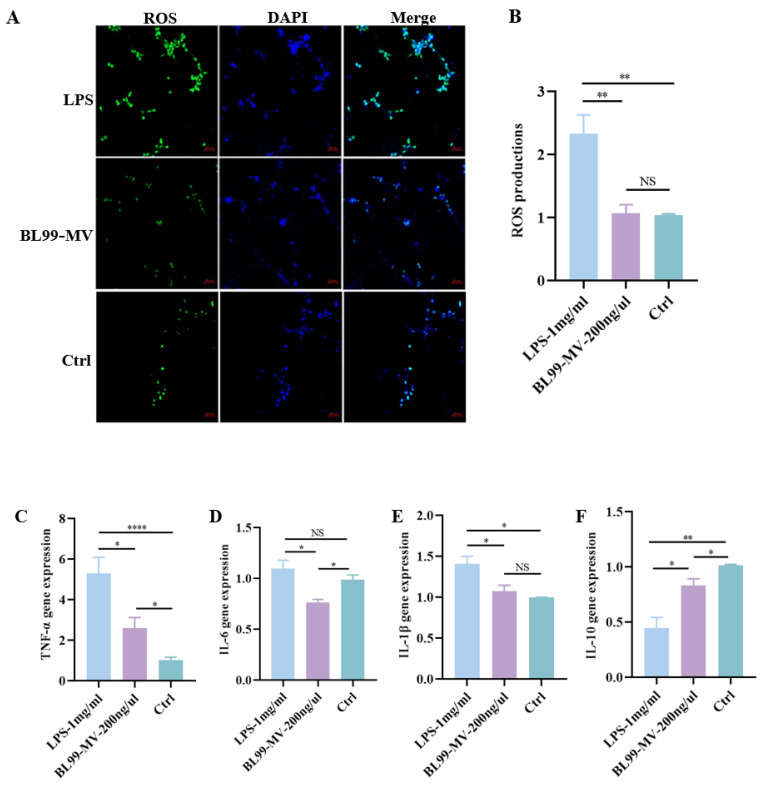
Effect of BL99-MV on anti-oxidative activation and anti-inflammation activation on HT22. (**A**,**B**) Fluorescence staining of ROS in HT22 exposed to LPS shows green fluorescence for ROS and blue fluorescence for DAPI. Scale Bar: 50 μm. (**C**–**F**) Relative quantification of inflammation factors level in the HT22 exposed to LPS (*n* = 6). Data are presented as the mean ± SEM, with three replicates per group (*n* = 3). Means marked with asterisks are significantly different (* *p* < 0.05; ** *p* < 0.01; **** *p* < 0.0001; NS *p* > 0.05).

To assess BL99-MV antioxidant capacity, we used the DCFH-DA probe to measure ROS activity in HT22. Confocal microscopy revealed that LPS significantly increased ROS levels in HT22, whereas BL99-MV significantly reduced ROS levels (*p* < 0.01) (Figure 5A). These findings were consistent with the results from qPCR analysis (Figure 5B).

To explore the anti-inflammatory properties of BL99-MV, we quantified TNF-α, IL-6, IL-1β, and IL-10 mRNA expression in HT22 using qPCR. The LPS group significantly increased TNF-α, IL-6, and IL-1β expression, while significantly inhibiting IL-10 expression in HT22 (*p* < 0.05). However, BL99-MV significantly reduced TNF-α, IL-6, and IL-1β expression, and significantly increased IL-10 expression (*p* < 0.05) (Figure 5C–F). These results indicate that BL99-MV possess both antioxidant and anti-inflammatory properties.

### 3.5. Transcriptomic Analysis

#### 3.5.1. Differential Expression Analysis Was Combined with Functional Enrichment

**Figure 6:**
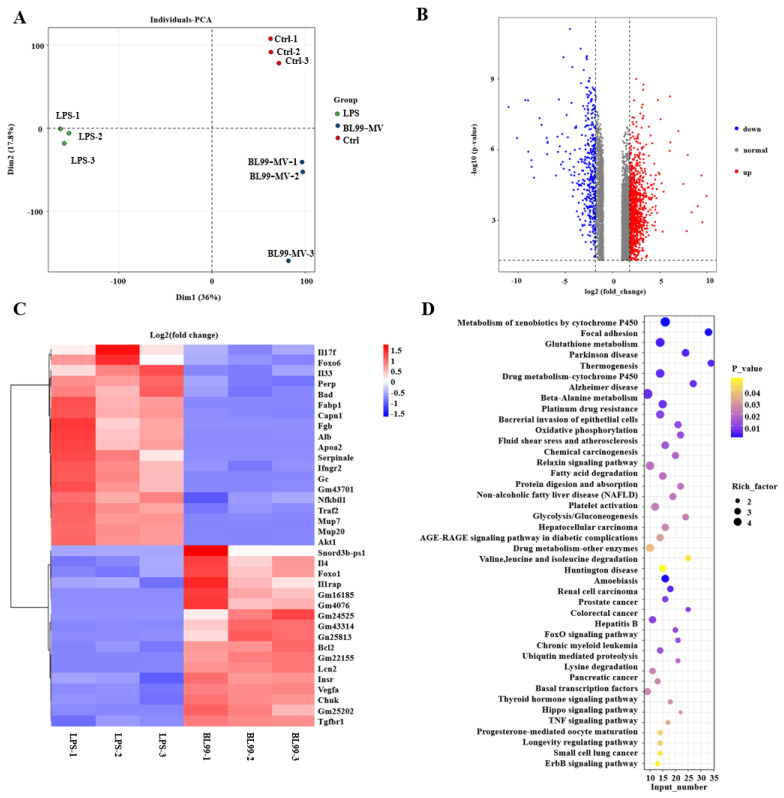
Differentially expressed mRNA. (**A**) PCA. (**B**) Fold change relative to statistical significance is shown. Red spots represent genes with significant upregulation, while blue spots indicate genes with significant downregulation. (**C**) Heat map of gene expression data. The heat plot illustrates 44 differentially expressed mRNA genes. Each column corresponds to a sample, while each row represents a specific gene. (**D**) Bubble plots depicting KEGG mRNA gene enrichment analysis.

To further investigate the effect of BL99-MV on HNF in HT22, we conducted transcriptome analysis using RNA sequencing (RNA-seq). The PCA revealed that the BL99-MV group was similar to the control group, with minimal variability between samples within each group (Figure 6A). Differential expression analysis identified 4127 genes in total, with 2549 genes significantly upregulated and 1578 genes downregulated in the BL99-MV group compared to the LPS group (Figure 6B). The volcano plot displayed these gene expression changes, while the heat map showed that samples from the BL99-MV group clustered together, reflecting similar gene expression patterns. Compared to the LPS group, BL99-MV significantly decreased the expression of IL17f, FoxO6, IL-33, Perp (P53), Bad, NFκBiI1 (NFκB), Traf2, and Akt1, while significantly increasing the expression of IL-4, FoxO1, Bcl2, Vegfa, and Chuk (IKKα) (*p* < 0.05) (Figure 6C). To reveal potential signaling pathways associated with BL99-MV-induced activation in HT22, we conducted KEGG enrichment analysis. With a significance threshold of *p* < 0.05 and |FC| ≥1.8, the differentially expressed genes were primarily enriched in 44 signaling pathways (Figure 6D). The most significantly enriched pathways included the Alzheimer’s disease signaling pathway and the FoxO signaling pathway.

#### 3.5.2. Validation by qRT-PCR

**Figure 7:**
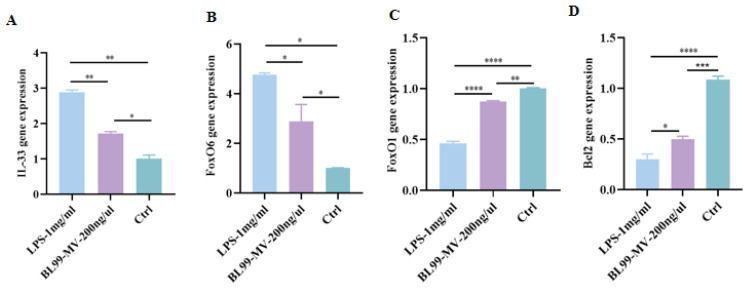
Effect of BL99-MV on the expression of IL-33, FoxO6, FoxO1, and Bcl2 in HT22 by qPCR. (**A**) qPCR of the mRNA expression of IL-33 in HT22. (**B**) qPCR of the mRNA expression of FoxO6 in HT22. (**C**) qPCR of the mRNA expression of FoxO1 in HT22. (**D**) qPCR of the mRNA expression of Bcl2 in HT22. Data are presented as the mean ± SEM, with three replicates per group (*n* = 3). Means marked with asterisks are significantly different (* *p* < 0.05; ** *p* < 0.01; *** *p* < 0.001; **** *p* < 0.0001; NS *p* > 0.05).

To validate the transcriptome results, we performed qPCR to analyze the expression levels of IL-33, FoxO6, FoxO1, and Bcl2. The qPCR results showed that BL99-MV significantly inhibited IL-33 and FoxO6 mRNA expression, while significantly promoting FoxO1 and Bcl2 mRNA expression in HT22 compared to the LPS group (*p* < 0.05) (Figure 7A–D). These trends were consistent with the RNA-seq data.

### 3.6. The BL99-MV Alleviated HNF Injury Against IL-33 by Regulating FoxO6/P53 Signaling

**Figure 8:**
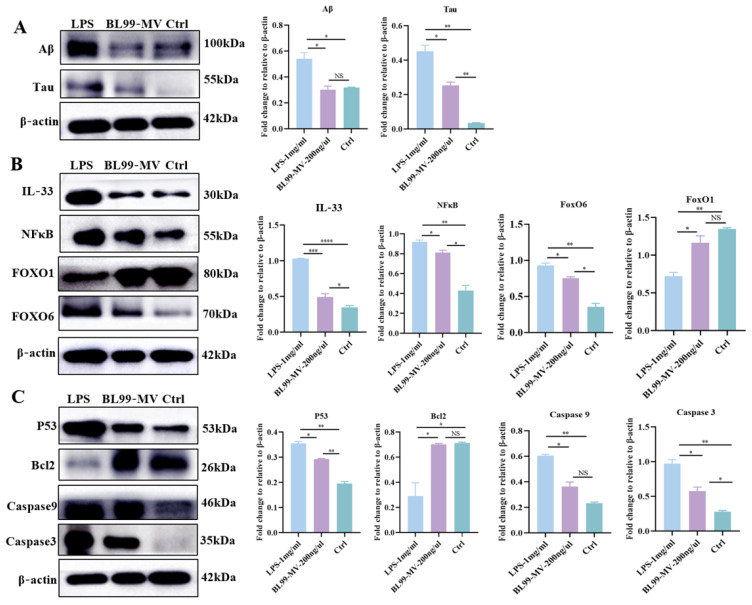
Effect of BL99-MV on the expression of Aβ, Tau, IL-33, NFκB, FoxO6, FoxO1, Caspase3, Caspase9, P53, and Bcl2 in HT22 by WB. (**A**) Blots of the protein expression of Aβ and Tau in HT22. (**B**) Blots of the protein expression of IL-33, NFκB, FoxO1, and FoxO6 in HT22. (**C**) Blots of the protein expression of P53, Bcl2, Caspase9 and Caspase3 in HT22. Data are presented as the mean ± SEM, with three replicates per group (*n* = 3). Means marked with asterisks are significantly different (* *p* < 0.05; ** *p* < 0.01; *** *p* < 0.001; **** *p* < 0.0001; NS *p* > 0.05).

To investigate the underlying mechanism of the anti-HNF effect of BL99-MV, we measured the expression of Aβ and Tau, which are key marker proteins related to the HNF signaling pathway using WB. The results indicated that Aβ and Tau proteins were highly elevated in HNF, and BL99-MV significantly decreased their expression. Given the central role of IL-33 in HNF, we measured its protein and mRNA expression levels using WB. The results indicated that BL99-MV significantly reduced IL-33 expression in HT22 compared to the LPS group, thereby modulating the downstream FoxO6/P53 pathways (Figure 8B). Furthermore, compared to the LPS group, BL99-MV significantly promoted the expression of FoxO1, while decreasing the expression of FoxO6 and its downstream apoptosis-related proteins (P53, Caspase9, and Caspase3) in HT22 (Figure 8C).

## 4. Discussion

Recent studies have revealed Gram-positive-derived MV are spherical membrane particles from 20 to 400 nm in diameter, such as *Akkermansia muciniphila*, *Bifidobacterium longum* [55], *Lactobacillus plantarum WCFS1* [56], *Lactobacillus rhamnosus GG*, and *Lactobacillus* [63]. The BL99, a member of the Gram-positive gut microbiota, has gained attention for its ability to regulate intestinal dysbiosis [40]. Our result showed BL99-MV to be 50–300 nm membrane vesicles with spherical membranes, which is concordant with previous reports [56]. The past research suggests that MV were isolated from strains cultured in MRS medium during the stationary growth phase. The *L. crispatus*-derived MV and *L. gasseri*-derived MV were isolated during the stationary growth phase in the MRS medium [50]. When *L. plantarum* reached a stationary phase, MV were isolated from the culture supernatants [64]. Based on previous studies, we isolated MV from BL99 supernatants in the stationary phase. Furthermore, the amounts of BL99-MV probably differ among bacteria in the growth stage. To our knowledge, this is the first study that describes the amounts of probiotic-derived MV by FC. Our result showed that BL99-MV can be stable in isolation during the period of bacterial growth, and the amount of BL99-MV was inconsistent. We observed that BL99 isolated the largest amounts of MV in the late stationary phase, however, the lowest amounts of BL99-MV were in the early stationary phase. Previous studies have shown that the protein content of MV was 1.19 μg/10^9^ particles. We describe Method 2 (ultracentrifugation combined with ultracentrifuge) with the aim of increasing the efficiency of isolating MV from BL99 supernatant, compared with previous protocols, which only used ultracentrifugation. BCA results showed that the protein concentration of MV by Method 2 was 1.34 μg/μL. The FC result showed that the amount of BL99-MV yielded by Method 2 was 248,797.

To date, a few studies have described only probiotics-mediated activity on neuroinflammation [32]. Gao et al. found that *Akkermansia muciniphila*-derived MV can attenuate cognitive dysfunction by suppressing microglia activation [65]. Mata Forsberg et al. reported that *Lactobacillus plantarum*-derived MV reduced neuronal apoptosis and improved neurological function [43]. *Lactobacilli*-derived MV are also able to dampen pro-inflammatory cytokine such as IFN-γ and IL-17A, and have an anti-inflammatory effect [66]. Based on previous studies, we question whether BL99-MV has neuroprotective potential and an anti-inflammatory effect. The BL99 is regarded as a beneficial bacterium with essential functions in alleviating intestinal inflammation, including colitis [39] and dyspepsia [40]. However, limited research has examined their effect on HNF. Our study, based on the gut–brain axis principle, explored how BL99-MV alleviated HNF. Previous studies demonstrated that *Lactobacillus murinus*-derived MV can be incorporated into macrophages [67]. *Lactobacillus Plantarum*-derived MV can be incorporated into neurons and significantly reduce neuronal apoptosis caused by ischemia [43]. Our result showed that BL99-MV can also be incorporated into HT22 and significantly increase HT22 vitality. The CCK8 result showed that, at a concentration of 200 ng/μL, it could significantly promote HT22 proliferation. Additionally, it has been demonstrated that BL99 exhibits strong anti-inflammatory effects in the intestine [39]. Our result showed that BL99-MV assumes a beneficial role in HNF by decreasing pro-inflammatory cytokine secretion and increasing anti-inflammatory cytokine secretion. Additionally, the activities of ROS were evaluated, which are key antioxidants involved in protecting nervous tissue. Our result showed that BL99-MV altered ROS expression in HT22, thereby inhibiting oxidative stress. These results illustrate that BL99-MV exerts a protective effect on LPS-induced cell damage by regulating inflammatory cytokines and oxidative stress.

In this study, the direct effects of BL99-MV on HT22 were first explored, revealing its potential beneficial impact on neuroinflammation. The RNA-seq experiment analyses identified several DEGs and pathways implicated in neuroinflammatory reactions between the BL99-MV and LPS groups. Notably, our findings suggest that Alzheimer’s disease and FoxO signaling pathways may be essential for these processes. The mRNA expression of IL-33 was significantly elevated in the LPS group, whereas BL99-MV treatment inhibited IL-33 expression. This observation was also corroborated at the mRNA and protein levels by qPCR and WB. Consistent with these findings, prior research reported that the mRNA and protein levels of IL-33 were elevated in the HMC3 cells under LPS-induced conditions [68]. IL-33 has been shown to be a novel biomarker of inflammation and has relevance to various infectious and inflammatory diseases [69]. Previous investigations have found that IL-33 is predominantly expressed in the hippocampus by neurons during cell injury [70]. During inflammatory progression, IL-33 is promptly released from the nuclei of necrotic cells and secreted into the extracellular space, which subsequently promotes NFκB nuclear translocation [71]. Its expression is regulated by signaling pathways, such as Aβ and Tau, as well as by transcription factors, like NFκB, FoxO1, and FoxO6. Both FoxO1 and FoxO6 are crucial transcription factors in the onset and progression of inflammatory disorders [72,73]. In the current research, we show evidence that the FoxO1 and FoxO6 transcription factors independently mediate HNF through IL-33 expression. Interestingly, IL-33 has been demonstrated to positively regulate the FoxO signaling pathways [73,74]. In agreement with previous studies, we observed that IL-33 upregulation inhibited FoxO1 expression while enhancing FoxO6 expression in neurons following LPS stimulation in HT22. This suggested a close connection between IL-33 and the FoxO6 signaling pathways. Overactivation of the FoxO6 signaling pathways has been implicated in LPS-induced HNF [22]. Our results further demonstrated significant upregulation of the pro-apoptotic genes P53, Caspase9, and Caspase3, alongside a downregulation of the anti-apoptotic gene Bcl2 in the LPS group. These findings indicate that LPS-induced activation of IL-33 may serve as an upstream mechanism driving pro-apoptotic factor activation, thereby reducing intracellular anti-apoptotic capacity.

**Figure 9:**
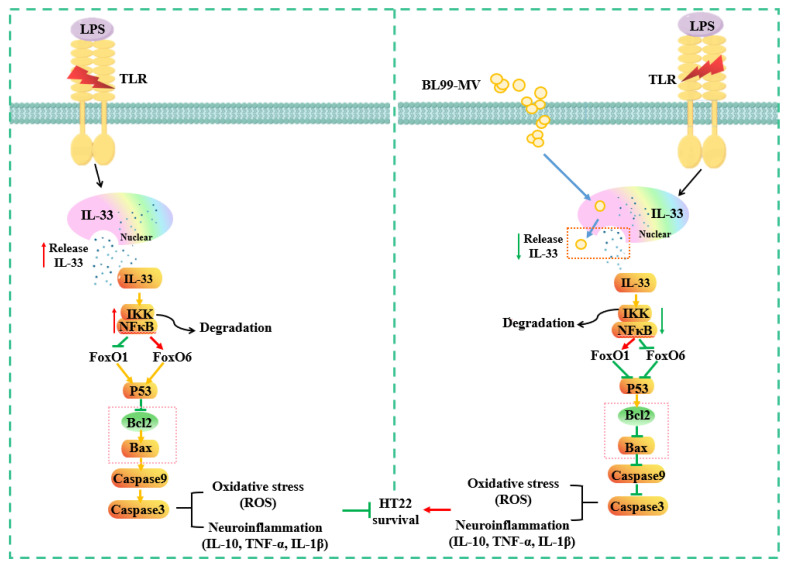
A proposed model for molecular mechanisms of the association between BL99-MV and HNF in HT22 cells.

Collectively, this study predicted that BL99-MV could induce a series of signal cascade reactions of FoxO6/P53 signaling pathways via IL-33 (Figure 9). However, as no related studies have been reported yet, further research is needed to explore these mechanisms. In conclusion, BL99-MV may offer neuroprotective effects against hippocampal neuroinflammation through multiple molecular pathways.

## 5. Conclusions

The findings of our study have revealed that MV can be released from BL99 stably, and that BL99-MV affect the release of IL-33 after being taken up by HT22 cells. When HNF occurred, BL99-MV had a marked effect on HT22 survival, improved the release of inflammatory factors, and reduced oxidative stress levels. A further study revealed 4127 differentially expressed genes, with 2549 genes upregulated and 1578 genes downregulated in the BL99-MV group compared to in the LPS group. BL99-MV decreased IL-33, FoxO6, and P53 expression, but increased FoxO1 expression compared with the LPS group. Then, BL99-MV affecting IL-33 release were confirmed to attenuate LPS-induced HNF via FoxO6/P53 signaling. These findings provide the evidence of a link between BL99-MV and FoxO6/P53 signaling in HNF, along with novel insights into the mechanism of MV.

## Figures and Tables

**Table 1:** Primer list.

Gene	Primer Sequences	Tm (°C)
Ki67	F: TGAGACCCGCAGCGTAAGG	53.1
	R: AGAGGCAGAGCGAAGGAAA	46.6
OPG	F: CCATACTCCGAGGGAATCA	46.6
	R: ACAGACCACCGTGTAGACA	46.8
TNF-α	F: ACTACCTCAACCGTTCCA	51
	R: GAGCTTCCCAGATCACAG	48.8
IL-6	F: AAGAAGAGTGGATGGGTTT	53.1
	R: GCGTTCCTGTCCTTGATAG	46.6
IL-1β	F: AGGAGCACCTCGGTATCA	51
	R: GTATTGCCATCAGCGTCC	51
IL-10	F: TGTGTGTTGGCTGAATTGT	51
	R: CTGCTCCTGGTGAGTCCTT	51
FoxO1	F: ACGCCGACCTCATCACCAA	48.8
	R: GGCACGCTCTTCACCATCC	46.6
Bcl2	F: ACATCCCAGCTTCACATAA	48.8
	R: CAATCCGACTCACCAATAC	53.1
Capn1	F: TACGCTGGCATCTTTCATT	50.2
	R: TTTAGCATAGGCTTTCTCC	50.2
Perp	F: GGTGGAGAAGAAGGAGTAT	50.2
	R: AAATGAAAGGAGTGGTGAG	45.6
Aβ	F: GCAGCGAGAAGAGCACTAA	51
	R: CAACGGGCAGCATACAAAC	51
Tau	F: CATCCCTACCAACACCGCC	55.3
	R: CTGCACCTTGCCACCTCCT	55.3
IL-33	F: CTGTTAGTTTTGTTTTGGA	48.8
	R: GTAGTAGCACCTGGTCTTG	51
NFκB	F: CAGAGGCGTGTATTAGGGG	53.1
	R: GTGCTGTCAGGGAGGAAGG	55.3
FoxO6	F: GTCCGCTACGTGCCCTACT	55.3
	R: GGGGTCTTGCCTGTCTTTC	51
P53	F: TGAGGTTCGTGTTTGTGCC	51
	R: CTCTCCATCAAGTGGTTTT	46.6
Caspase9	F: AACGACCTGACTGCCAAGA	51
	R: GAGAGGATGACCACCACAA	51
Caspase3	F: GGGACTGATGAGGAGATGG	53.1
	R: GCTGCAAAGGGACTGGATG	53.1

## Data Availability

We permit unrestricted use, distribution, and reproduction in any medium, provided the original work is properly cited. Sequencing data generated in this study were deposited in the Sequence Read Archive (SRA) (https://www.ncbi.nlm.nih.gov/sra/, accessed on 1 October 2024) with the BioProject ID: PRJNA1172725.

## Supplementary Materials

The following supporting information can be downloaded at: https://www.mdpi.com/article/10.3390/nu16213586/s1, Figure S1: The protein concentration of MV by BCA. Data are presented as the mean ± SEM, with three replicates per group (*n* = 3).
