# Supplementary material for: Bifidobacterium lactis-Derived Vesicles Attenuate Hippocampal Neuroinflammation by Targeting IL-33 to Regulate FoxO6/P53 Signaling
Source: Nutrients. 2024 Oct 22;16(21):3586. doi: 10.3390/nu16213586 (PMC11547434; doi:10.3390/nu16213586)
Supplement: Supplementary file 1 [file nutrients-16-03586-s001.zip › nutrients-3229450-supplementary.pdf]

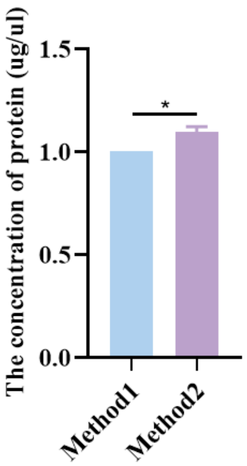

Figure S1. The protein concentration of MV by BCA. Data are presented as the mean ± SEM, with three replicates per group (n = 3). Means marked with asterisks are significantly different (* $p < 0.05$).
